# Overlapping Autoimmune Diseases: A Case Report and Review of Eosinophilic Granulomatosis With Polyangiitis and Mixed Connective Tissue Disease

**DOI:** 10.7759/cureus.43584

**Published:** 2023-08-16

**Authors:** Muaz Shafique Ur Rehman, Muhammad Subhan, Shaina Gulraiz, Ruqiya Bibi, Muhammad Waqas, Anzal Ishfaq, Saad Siddiq Muhammad, Abubakar Gapizov, Faris Fayyaz

**Affiliations:** 1 Internal Medicine, Jinnah Hospital, Lahore, PAK; 2 Internal Medicine, Jinnah Hospital, Allama Iqbal Medical College, Lahore, PAK; 3 Internal Medicine, Al Barkat Health Care and Collection Centre, Lahore, PAK; 4 Editorial Department, International Journal of Clinical and Molecular Oncology, Lahore, PAK; 5 Emergency Medicine, Allama Iqbal Medical College, Lahore, PAK; 6 Medicine and Surgery, Jinnah Hospital, Lahore, PAK; 7 Internal Medicine, Allama Iqbal Medical College, Lahore, PAK; 8 Internal Medicine, Jinnah Sindh Medical University, Karachi, PAK; 9 Internal Medicine, Mayo Hospital, Lahore, PAK; 10 Internal Medicine, Inner Mongolia University for Nationalities, Tongliao, CHN; 11 General Surgery, American University of Antigua, Osbourn, ATG; 12 Surgery, Dow University of Health Sciences, Karachi, PAK

**Keywords:** churg-strauss syndrome, vasculitis, systemic lupus erythematosus, dermatopathology, polymyositis, sle pathogenesis, antineutrophil cytoplasmic antibody (anca) associated vasculitis (aav), panca/mpo (myeloperoxidase)-positive microscopic polyangiitis (mpa), granulomatosis with polyangiitis (gpa), eosinophilic granulomatosis with polyangiitis (egpa)

## Abstract

We describe a rare case of concurrent eosinophilic granulomatosis with polyangiitis and mixed connective tissue disease in a 27-year-old man who presented with pulmonary, renal, cardiac, and skin manifestations. We confirmed the diagnosis based on clinical, histopathological, and serological criteria. We treated the patient with corticosteroids, methotrexate, cyclophosphamide, and hydroxychloroquine, achieving early remission. The coexistence of both conditions in the same patient is extremely rare and has only been reported in a few cases worldwide. We also review the literature on these two rare autoimmune diseases' coexistence, pathogenesis, diagnosis, and management. Our case emphasizes recognizing overlapping autoimmune conditions in patients with complex clinical features and employing a comprehensive diagnostic approach and tailored treatment strategies.

Further research is needed to understand these patients' epidemiology, prognosis, and optimal therapy. Early diagnosis and aggressive immunosuppression are crucial for achieving remission and preventing organ damage. We also identified the knowledge gaps and research needs in this field.

## Introduction

Eosinophilic granulomatosis with polyangiitis (EGPA), also called Churg-Strauss syndrome (CSS), is a rare autoimmune systemic vasculitis that affects small-to-medium vessels, and multiple organs, including the paranasal sinuses, lungs, skin, nerves, heart, eye, and kidneys [1]. Mixed connective tissue disease (MCTD) is another rare autoimmune disorder that presents with mixed clinical manifestations of systemic lupus erythematosus (SLE), scleroderma (SD), and polymyositis (PM)/dermatomyositis (DM) [2]. Both diseases can present with multisystemic involvement and have overlapping clinical features, making the differential diagnosis challenging [1,2]. Researchers estimate that EGPA affects between two and 38 cases per million people worldwide, with a bimodal age distribution and no gender preference [3]. The prevalence of MCTD is estimated to be between 3.8 and 4.8 cases per 100,000 people worldwide, predominantly in females, and occurs in the second to third decades of life [4]. The pathogenesis of both diseases is not fully known, but it is believed to involve a complex interplay of genetic, environmental, and immunological factors [5]. EGPA is characterized by asthma, elevated immunoglobulin E (IgE), eosinophilia, and antineutrophil cytoplasmic antibodies (ANCAs) [5]. MCTD is characterized by high titers of anti-U1 ribonucleoprotein (U1-RNP) antibodies and clinical features of SLE, SD, and PM/DM [6]. The diagnosis of EGPA and MCTD usually involves a combination of clinical criteria, laboratory investigations, and imaging studies [7]. The management of EGPA and MCTD usually consists of various immunosuppressive agents (corticosteroids, methotrexate, cyclophosphamide, and hydroxychloroquine) and supportive care [8]. However, surveillance for these patients must be better established and may vary depending on the disease activity, organ involvement, and response to therapy [8,9]. In this case report, we present a rare case of a 27-year-old man with concurrent EGPA and MCTD who presented with severe joint pain, stiffness, numbness, tingling sensations in hands and feet, weight loss, fatigue, night sweats, skin rashes, rhinosinusitis, asthma, eosinophilia, and peripheral neuropathy. We also review the literature on these two rare autoimmune diseases' coexistence, pathogenesis, diagnosis, and management. Our case report highlights the importance of accurate differential diagnosis in patients with complex clinical features and the need for further research to understand better the epidemiology, prognosis, and optimal therapy for these patients.

## Case presentation

**Figure 1:**
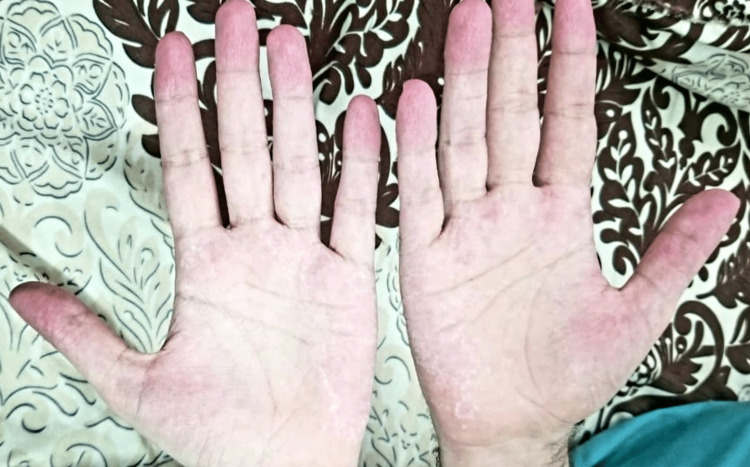
Red swollen fingertips with palmer skin changes

We report a rare case of a 27-year-old man who presented with severe pain and stiffness in the joints of his hands and feet. He also reported experiencing pain in his left upper and lower limbs, accompanied by tingling sensations like pins and needles, fatigue, unexplained weight loss, night sweats, itchy skin rashes, heartburn, and cough with bloody sputum. He had a long-term history of nasal allergies and asthma from four and three years, respectively, for which he took steroids nasal spray, montelukast sodium, and inhaled corticosteroids. He also partially followed an immunotherapy course to treat allergies but discontinued it due to a lack of noticeable improvement. He underwent functional endoscopic sinus surgery for sinonasal polyps, and the histopathology of the biopsy tissue revealed eosinophil-enriched inflammatory infiltration. On general physical examination, we observed oropharyngeal erythema, red swollen fingertips with erythrodermic palmer skin eruptions, Raynaud's phenomenon, and blanching pin-pointed red spots on the body (Figure 1).

**Figure 2:**
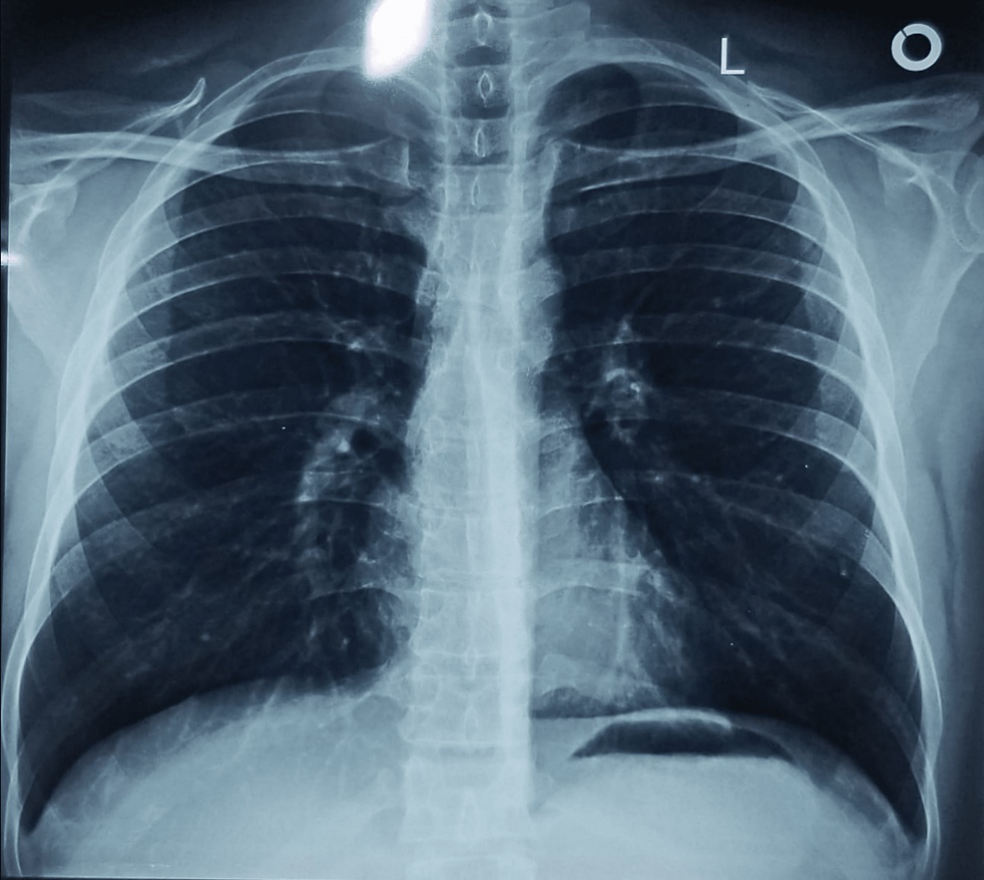
Chest X-ray showing bilateral pulmonary hyperinflation with perihilar infiltrates

On chest auscultation, we heard a bilateral wheeze. Central nervous system examination revealed reduced sensations in the left hand's thumb, index finger, and left leg calf region, suggesting peripheral neuropathy (PN; mononeuritis multiplex). All other general and systemic examination was normal. Preliminary laboratory investigations revealed elevated white blood cells (18.4 × 10^9/L), normal platelets (201 × 10^9/L), and elevated eosinophils (13%) in complete blood count (CBC), elevated erythrocyte sedimentation rate (ESR; 44 mm/hr), elevated C-reactive protein (CRP; 24.8 mg/L), low vitamin D3 (20 ng/ml), increased IgE levels (181.7 IU/ml), normal liver function tests (LFTs) with bilirubin at 0.4 mg/dl, alanine aminotransferase at 34 U/L, and aspartate aminotransferase at 23 U/L, normal renal function tests (RFTs) with urea at 19 mg/dl and creatinine at 0.7 mg/dl, and urine complete examination (UCE) showed trace proteins. On chest X-ray, we saw bilateral hyperinflation with perihilar lung infiltration, suggesting pulmonary involvement (Figure 2).

**Figure 3:**
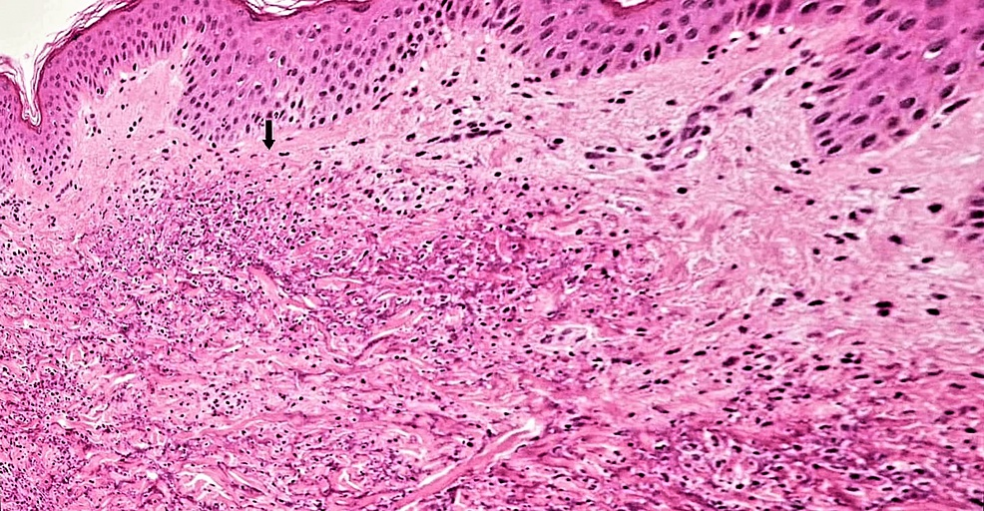
Biopsy of skin showing histological findings of eosinophilic tissue infiltrates

After suspecting an autoimmune disease, we consulted a rheumatologist, who advised autoimmune screening tests. The autoimmune test profile showed positive rheumatoid factor (RF) and antinuclear antibodies (ANA) with a fine cytoplasmic speckled pattern. Anti-cyclic citrullinated peptide (ACCP) antibodies, anti-double-stranded deoxyribonucleic acid (anti-dsDNA) antibodies, cytoplasmic anti-neutrophil cytoplasmic antibodies (C-ANCA), and perinuclear anti-neutrophil cytoplasmic antibodies (P-ANCA) were negative. We made a clinical diagnosis of EGPA that was consistent with the criteria of the American College of Rheumatology [10], which we further confirmed with a skin biopsy showing tissue infiltration with eosinophils (Figure 3).

Later, the extractable nuclear antigen (ENA) profile results showed positive U1-RNP and anti-Ku antibodies, combined with positive ANA and clinical presentation, suggesting the diagnosis of MCTD based on the Alarcon-Segovia criteria [11]. The conclusive diagnosis indicated that the patient had concurrent features of EGPA and MCTD. We started the patient's treatment with high-dose prednisolone 60 mg daily, which we gradually tapered off over several weeks. In addition to prednisolone, we prescribed methotrexate (MTX) (15 mg weekly) and hydroxychloroquine (400 mg twice daily in divided doses), later adjusted to a fixed dosage of 200 mg per day. We also gave cyclophosphamide (CYC) intravenously at 0.6-1 g/m2 every three to four weeks and mesna in the same dose as CYC. We gave montelukast sodium, a corticosteroids inhaler, cetirizine, and omeprazole to manage asthma, nasal allergy, and heartburn symptoms. We also added supplements like vitamin D3 and other multivitamins. The patient responded well to the treatment and achieved early remission. Follow-up after two months showed a much-improved patient condition, and laboratory tests showed normal CBC, ESR, CRP, LFTs, RFTs, and UCE.

## Discussion

This report presented a rare case of a patient with overlapping autoimmune diseases, namely, EGPA and MCTD. Overlapping autoimmune disorders is a general term that refers to the coexistence of two or more autoimmune diseases in the same patient [11,12]. Overlap syndrome is a more specific term that describes a condition where a patient has features of two or more well-defined autoimmune diseases without meeting the full diagnostic criteria for any single disease [11,12]. For example, MCTD is an overlap syndrome that combines features of SLE, Sjögren's syndrome (SS), and PM/DM [2]. Therefore, overlap syndrome is a subset of overlapping autoimmune diseases. We discussed the challenges in diagnosing these conditions and the importance of differentiating them for proper management. We also reviewed the clinical, laboratory, and histopathological aspects of EGPA and MCTD and compared them with recent studies.

**Figure 4:**
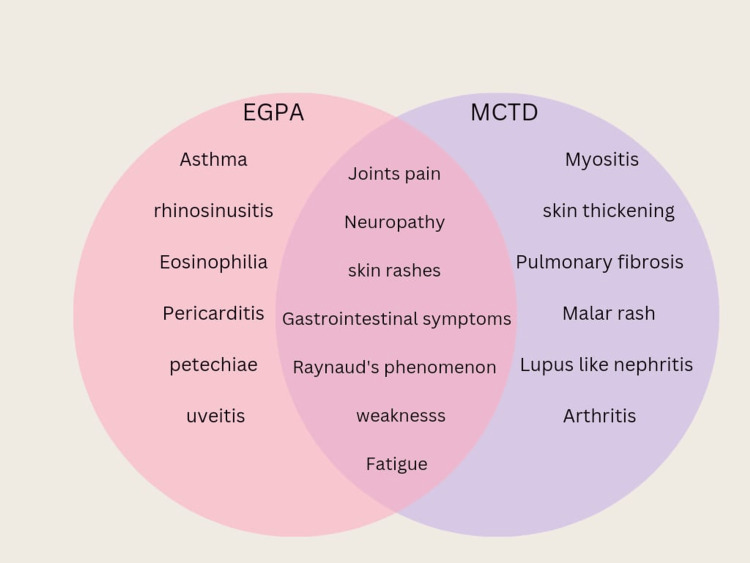
Clinical signs and symptoms of EGPA and MCTD EGPA: eosinophilic granulomatosis with polyangiitis; MCTD: mixed connective tissue disease.

EGPA is a form of ANCA-associated vasculitis (AAV) that causes damage to small and medium-sized blood vessels by inflammation and eosinophilic infiltration in various organs [3]. The main symptoms of EGPA are rhinosinusitis, asthma, eosinophilia, and vasculitic organ involvement [3]. The pathogenesis of EGPA involves activating eosinophils and releasing pro-inflammatory cytokines, which may lead to vascular occlusion and tissue damage [3]. On the other hand, MCTD is an autoimmune disorder that combines features of SLE, SS, and PM/DM [2]. It was first described by Sharp et al. in 1972 [2]. MCTD is associated with high levels of U1-RNP, which are very specific to this disease [6]. The clinical presentation of EGPA and MCTD can overlap, as both diseases can affect multiple organs [6,7]. EGPA typically has three phases: prodromal, eosinophilic, and vasculitic [10]. The prodromal phase is characterized by nonspecific rhinosinusitis symptoms with nasal polyposis and asthma [10]. The eosinophilic step involves elevated eosinophils in the blood and tissues, which may cause joint pain [10]. The vasculitic phase is marked by blood vasculopathy and organ-specific symptoms affecting the heart, kidneys, gastrointestinal tract, eyes, and nervous system [10]. MCTD, meanwhile, can manifest with various symptoms, such as skin rashes, arthritis, gastrointestinal disturbance, Raynaud's phenomenon, myositis, interstitial lung disease (ILD), and renal involvement [2]. However, these clinical features are not specific to MCTD and may vary over time [11]. Major clinical features of EGPA and MCTD are depicted in the Venn diagram in Figure 4 [2,10,11].

The age and gender distribution of EGPA and MCTD suggest that these diseases may have different risk factors and mechanisms, such as hormonal, genetic, environmental, and chronic factors, that may trigger or worsen EGPA or MCTD in susceptible individuals [7]. Some of the risk factors associated with EGPA are shown in Table 1 [7,8].

**Table 1:** Comparison of risk factors for EGPA and MCTD EGPA: eosinophilic granulomatosis with polyangiitis; MCTD: mixed connective tissue disease.

Risk factor	EGPA	MCTD
Female sex	No clear association	More common in women
Age	It usually affects adults, with a mean age of onset of 48 years	More common in women. It usually affects young adults, with a mean age of onset of 37 years
Genetic factors	Some genes or gene variants may be associated with EGPA, such as HLA-DQ2.5, HLA-DRB1*04:04, and IL10	Some genes or gene variants may predispose people to develop MCTD, such as HLA-DR4, tumor necrosis factor-alpha, and STAT4
Environmental factors	Some environmental factors may trigger or worsen EGPA and MCTD, such as infections, stress, emotional distress, cold exposure, or switching or stopping medications	-
Asthma/allergies	Almost all people with EGPA have a history of asthma and allergies	Not a typical feature of MCTD

Laboratory investigations are vital in differentiating EGPA from MCTD [2,3]. In EGPA, eosinophilia is a crucial feature, and ANCA can be detected in about half of the patients [3]. Moreover, elevated levels of serum IgE may support the diagnosis of EGPA [3]. In contrast, MCTD is characterized by high levels of U1-RNP, which can help distinguish it from other connective tissue diseases (CTDs) [2]. Several studies have explored the pathogenesis, etiology, risk factors, genetic associations, and management guidelines for EGPA and MCTD. Some studies have also investigated the role of autoimmune antibodies in these diseases. For example, Pyo et al. evaluated the performance of the 2021 American College of Rheumatology/European Alliance of Associations for Rheumatology (ACR/EULAR) criteria for diagnosing AAV in a group of Korean patients and compared them with the older criteria [12]. They also examined ANCA's association with different AAV types, such as EGPA, granulomatosis with polyangiitis (GPA), and microscopic polyangiitis (MPA) [12]. They found that the 2021 ACR/EULAR criteria had higher sensitivity and specificity than the older criteria and that ANCA was more prevalent in GPA and MPA than EGPA [12]. This study can be compared with another study on MCTD by Menor Almagro et al. [13]. They conducted a cross-sectional study with 1,002 patients who underwent ANA testing in Spain between 2009 and 2011 [13]. They found that 293 patients (29.2%) had positive ANA results and that 105 patients (10.5%) had a diagnosis of MCTD [13]. They also found that higher ANA titers were associated with a higher probability of having MCTD, especially SLE and SS [13]. Thus, both studies showed an association of autoimmune antibodies with EGPA and MCTD [12,13]. Many systemic features are common in vasculitic and autoimmune diseases like EGPA, GPA, MPA, SLE, and SS [14]. One of these common features is PN [14]. Bischof et al. analyzed data from 666 patients with AAV and found that 19.5% of AAV patients had PN, which was more common in GPA than in MPA and EGPA [14]. The relative risks (RR) for PN in GPA compared with MPA and EGPA were 2.08 with a 95% CI of 1.45-2.98 and 2.33 with a 95% CI of 1.32-4, respectively [14]. Hence, GPA patients were more likely to have PN than MPA or EGPA patients [14]. EGPA and MCTD can affect organ systems like the lungs [7,8]. Hasegawa et al. conducted a retrospective cohort study with 1,050 hospitalized patients in Japan with EGPA between 2010 and 2012. They found that 20.8% of EGPA patients died during hospitalization and that lung involvement was an independent risk factor for in-hospital mortality [7]. RR for in-hospital mortality in EGPA patients with lung involvement compared with those without was 1.69 with a 95% CI of 1.31-2.18 [7]. So, EGPA patients with lung involvement were likelier to die during hospitalization than those without lung involvement [7]. As mentioned above, the study can be compared with the meta-analysis of Joy et al. [15]. They performed a mixed meta-analysis and reviewed 96 studies with 16,396 patients with CTD and ILD [15]. They found that the pooled prevalence of ILD was 29% in CTD patients, with the highest majority in SS at 47% and MCTD at 56% [15]. RR for ILD in different CTD subtypes compared with rheumatoid arthritis (RA) were 1.62 with a 95% CI of 1.35-1.94 for SS, 1.41 with a 95% CI of 1.11-1.79 for idiopathic inflammatory myositis (IIM), 0.59 with a 95% CI of 0.45-0.77 for primary Sjögren's syndrome (PSS), 1.92 with a 95% CI of 1.28-2.88 for MCTD, and 0.21 with a 95% CI of 0.14-0.31 for SLE [15]. Thus, SS, IIM, and MCTD patients were more likely to have ILD than RA patients, while PSS and SLE patients were less likely to have ILD than RA patients [15]. Zhao et al. conducted a case-control study with 1,000 AAV patients and 2,000 healthy controls in China. They found that smoking, silica exposure, infections, and drugs were linked with an elevated risk of AAV [16]. The RR for these factors were 1.75 with a 95% CI of 1.41-2.17 for smoking, 2.01 with a 95% CI of 1.63-2.48 for silica exposure, 2.13 with a 95% CI of 1.73-2.62 for infections, and 1.86 with a 95% CI of 1.51-2.29 for drugs [16]. This means AAV patients were more likely to have these risk factors than healthy controls [16]. Clinical research and studies related to EGPA and MCTD are relatively scarce. The findings and interpretations of the two latest studies are summarized in Table 2.

**Table 2:** Summary of selected clinical studies related to EGPA and MCTD and their results EGPA: eosinophilic granulomatosis with polyangiitis; MCTD: mixed connective tissue disease; ACR: American College of Rheumatology; EULAR: European Alliance of Associations for Rheumatology; ANCA: antineutrophil cytoplasmic antibody; AAV: ANCA-associated vasculitis; GPA: granulomatosis with polyangiitis; MPA: microscopic polyangiitis; ILD: interstitial lung disease; CTD: connective tissue disease; SS: Sjögren's syndrome; PN: peripheral neuropathy; ANA: antinuclear antibodies; SLE: systemic lupus erythematosus.

References	Publisher	Year of publication	Study design	Cases	Controls	Results
Pyo et al. [12]	Yonsei Med J	2023	Retrospective cohort study	1000	No control group	The 2021 ACR/EULAR criteria had higher sensitivity and specificity than the older criteria for diagnosing AAV. ANCA was more prevalent in GPA and MPA than EGPA.
Joy et al. [15]	Eur Respir Rev	2023	Systematic review and meta-analysis	16,396	No control group	ILD was prevalent (29%) and variable in CTD patients, with SS and MCTD having the highest rates (47% and 56%).
Zhao et al. [16]	Front Immunol	2020	Case-control study	1000	2000	Smoking, silica exposure, infections, and drugs were associated with an increased risk of AAV.
Bischof et al. [14]	Neurol Neuroimmunol Neuroinflamm	2019	Retrospective cohort study	666	No control group	PN was common (19.5%) in AAV patients and more frequent in GPA than in MPA and EGPA.
Menor Almagro et al. [13]	Reumatol Clin	2017	Cross-sectional study	293	709	ANA positivity was common (29.2%) and associated with MCTD (10.5%), especially SLE and SS. Higher ANA titers increased the probability of having MCTD.

The accurate diagnosis of EGPA vs. MCTD is crucial for proper management [2,3]. Biopsy and histopathology of affected tissues can provide additional insights into the differentiation of EGPA and MCTD. In our case, we confirmed the diagnosis based on clinical, histopathological, and serological criteria. We treated the patient with corticosteroids (prednisolone), methotrexate (MTX), cyclophosphamide (CYC), and hydroxychloroquine (HCQ), achieving early remission. Maritati et al. compared the efficacy and safety of MTX and CYC for remission maintenance in AAV. Remission was induced with CYC, and then patients were randomized to receive MTX or CYC as maintenance therapy [17]. Results depicted no solid statistical association between these two groups regarding relapse frequency or relapse-free survival one year or two years after randomization [17]. The guideline for treatment when features of two different diseases are present is not fixed or universal but rather individualized and multidisciplinary [18]. The treatment should be based on a comprehensive diagnostic approach that considers each patient's predominant clinical presentation, histological findings, and laboratory finding [18]. The treatment should also be monitored and adjusted according to the response and tolerance of each patient [18]. Treatment options for EGPA involve a combination of glucocorticoids, immunosuppressive agents, and biological antibodies, such as prednisone, MTX, CYC, or mepolizumab, aimed at controlling the inflammatory response and preventing organ damage [2,10]. MCTD, on the other hand, also involves management with glucocorticoids (prednisolone) and immunosuppressants (hydroxychloroquine) and may require a multidisciplinary approach involving various specialists, such as rheumatologists, pulmonologists, dermatologists, and gastroenterologists to manage specific symptoms and complications of the disease [2,8].

Limitations

This case report and review have some limitations, such as lack of generalizability from a single case, limited number and quality of studies reviewed, potential publication and heterogeneity bias, and need for more research to validate and update our findings.

## Conclusions

This report illustrates a rare case of a patient who had both EGPA and MCTD, two uncommon autoimmune disorders that can have overlapping clinical features. Diagnosing these conditions was challenging and required a comprehensive evaluation of the patient's history, symptoms, laboratory tests, histopathology, and expert consultations. The patient was treated with steroids (prednisolone) and immunosuppressants (CYC), hydroxychloroquine, and MTX, which improved his condition. More research is needed to elucidate the pathogenesis, etiology, risk factors, genetic associations, and management guidelines for EGPA and MCTD and to develop better diagnostic tools and therapeutic strategies for these diseases.
